# Treatment of children with refractory/relapse high risk langerhans cell histiocytosis with the combination of cytarabine, vindesine and prednisone

**DOI:** 10.1186/s12887-023-04465-5

**Published:** 2024-01-03

**Authors:** Wenqian Wang, Jian Ge, Honghao Ma, Hongyun Lian, Lei Cui, Yunze Zhao, Zhigang Li, Tianyou Wang, Rui Zhang

**Affiliations:** 1 Hematology Center, Beijing Key Laboratory of Pediatric Hematology Oncology, National Key Discipline of Pediatrics, Key Laboratory of Major Diseases in Children, Beijing Children’s Hospital, Capital Medical University, Ministry of Education, Capital Medical University, National Center for Children’s Health, Beijing, 100045 China; 2 Hematologic Disease Laboratory, Beijing Pediatric Research Institute,Hematology Center, Beijing Key Laboratory of Pediatric Hematology Oncology, Key Laboratory of Major Disease in Children, Beijing Children’s Hospital, National Key Discipline of Pediatrics,Capital Medical University, Ministry of Education, Capital Medical University, National Center for Children’s Health, Beijing, 100045 China

**Keywords:** Langerhans cell histiocytosis, Refractory, Relapse, Risk organ, Cytarabine

## Abstract

**Background:**

The patients with multisystem and risk organ involvement Langerhans cell histiocytosis (MS-RO + LCH) have poor prognosis. The patients with MS-LCH who failed front-line therapy have a high mortality rate and the standard salvage treatment has not been established. The combination of cytarabine (Ara-c), vincristine (VCR) and prednisone might be effective for refractory/relapse MS-RO + LCH, with low toxicity.

**Methods:**

We retrospectively analyzed pediatric refractory/relapse MS-RO + LCH patients treated with the low-dose Ara-c (100mg/m^2^/d×5days) or high-dose Ara-c (500mg/m^2^/d×5days) combined with vindesine (VDS) and prednisone in a single center. The efficacy, outcomes and adverse events were analyzed.

**Results:**

From January 2013 to December 2016, 13 patients receiving the low-dose Ara-c chemotherapy (LAC) and 7 patients receiving the high-dose Ara-c chemotherapy (HAC) were included in the study. 11 (84.6%) of the 13 patients treated with the LAC regimen and 6 (85.7%) of the 7 patients treated with the HAC regimen had response after four courses of the therapy. All patients in the study were alive during follow-up and the 3-year event-free survival rate (EFS) was 53.7% and 85.7% in the LAC and HAC groups. The most frequent adverse event was Grade 1/2 myelosuppression, which was observed in 38.5% (5/13) and 42.9% (3/7) of the patients receiving the LAC and HAC regimen.

**Conclusions:**

A combination of Ara-c, VDS and prednisone was effective and safe for some patients with refractory/relapse MS-RO + LCH. The high-dose Ara-c regimen was associated with a numerically higher EFS rate.

## Background

LCH is an inflammatory myeloid neoplastic disorder characterized by accumulation of CD1a and CD207 + dendritic cells. Its clinical manifestations range from self-limited lesions to widespread disseminated disease with life-threatening organ dysfunction [1]. The patients with multisystem LCH, especially with risk organs involvement (MS-RO + LCH), including liver, spleen and hematological system, have a higher risk of disease-related mortality than the patients with single system involvement [2]. Severe liver involvement can develop to some permanent sequelae including sclerosing cholangitis and cirrhosis of liver, which commonly evolve to end-stage liver failure [3]. The involvement of hematological system presenting as cytopenia and hemophagocytic lymphohistiocytosis (HLH) usually occur in very young children, with a high mortality rate [3, 4]. 29% of the patients with MS-RO + LCH were refractory to the VBL-steroid based front-line therapy and 27% of them experienced reactivation in the first two years [5]. Patients with MS-LCH who failed the front-line therapy had extremely poor prognosis, with an overall 3-year survival rate less than 20% [6, 7]. Thus, an effective salvage regimen is essential to improve the survival rate of the patients with refractory/relapse MS-RO + LCH.

However, the standard salvage treatment has not been established. Cytarabine (Ara-c) and cladribine (2-CDA) was promising as a salvage therapy for refractory/relapse MS-RO + LCH patients [8]. The combination of different doses of Ara-c and 2-CDA has been demonstrated effective for the treatment of refractory MS-RO + LCH patients. A combination of the high-dose Ara-c (1 g/m^2^/d×5 days) and 2-CDA (9mg/m^2^/d×5 days) had high toxicity, with the mortality rate of 15% [9]. A low-dose combination of Ara-c (100mg/m^2^/d×4days) and 2-CDA (5mg/m^2^/d×5days) regimen, had much less toxicity than the high-dose regimen, but all patients still experienced Grade III or IV myelosuppression [10]. In addition of the combination of Ara-c and 2-CDA regimen, the low-dose Ara-c (100mg/m^2^/d×5days) combined with vincristine (VCR) and prednisolone was effective in newly diagnosed LCH patients, with a response rate of 76% in MS-RO + patients [11]. The low-dose Ara-c with or without VCR and prednisone was also used in recurrent LCH patients, 67% (4/6) of the MS-RO + LCH patients had disease improvement, with lower toxicity and the 3-year overall survival rate (OS) of 100% [12]. Therefore, the combination of Ara-c, VCR and prednisone might be effective for refractory/relapse MS-RO + LCH patients. However, the number of cases of refractory/relapse MS-RO + LCH patients treated with Ara-c combined with VCR and prednisone was small in previous studies. Additionally, the dose of Ara-c varied from 100 to 170mg/m^2^/d in the regimen, and there was a lack of studies on the use of a higher dose of Ara-c combined with VCR and prednisone in the treatment of LCH patients.

In this study, we retrospectively analyzed efficacy, long term outcomes and adverse events of refractory/relapse MS-OR + LCH patients treated with the low-dose (100 mg/m^2^/d×5 day) or high-dose (500 mg/m^2^/d×5 day) Ara-c combined with vindesine (VDS) and prednisone.

## Methods

### Study design and patients

We reviewed the patients (age < 18 years) treated with Ara-c combined with VDS and prednisone for LCH at Beijing Children Hospital from January 2013 to December 2016. The inclusion criteria were positive staining of CD1a and /or Langerin (CD207) of biopsy tissue, at least one RO involvement, refractory to or relapsed after the first-line therapy. The first-line therapy was a VDS-prednisone combination treatment. VBL is not available in China and VDS has less neurotoxicity than VCR [13].Therefore, we used VDS to treat patients with LCH in our center. The induction treatment for 6–12 weeks was as follows: VDS, 3 mg/m^2^/day, once a week for 6 weeks; prednisone 40 mg/m^2^/day, daily for 4 weeks, afterward a weekly reduction for 2 weeks. The maintenance therapy consisted of VDS (3 mg/m^2^/day, every 3 weeks), prednisone (40 mg/m^2^/day, Day 1–5, every 3 weeks), and 6-mercaptopurine (50 mg/m^2^/day, daily). The overall duration of the first-line therapy was 12 months [14]. Refractory was defined as one or more ROs showed no improvement or disease progression after first-line therapy [15]. Relapse was defined as the reappearance of signs and symptoms of active disease after either complete disease resolution or a period of disease control that persisted for 3 months on maintenance therapy [16]. The exclusion criteria was nonstandard chemotherapy courses. Nonstandard chemotherapy courses included chemotherapy withdraw, switching to other therapy or receiving additional LCH-specific therapy before completing the standard treatment course, and these were not due to chemotherapy toxicity or unsatisfactory response. This study was approved by the Ethics Committee of Beijing Children’s Hospital. Informed consent to the treatment regimens was signed by the patients’ legal guardians.

### Therapeutic regimens and response assessment

There were two different doses of induction treatment regimens. The low-dose Ara-c chemotherapy regimen (LAC) consisted of Ara-c (100 mg/m^2^/day IV guttae, Day 1–5), VDS (3 mg/m^2^/day IV bolus, Day 1) and dexamethasone (6 mg/m^2^ /day, IV or orally, Day 1–5), once every 3 weeks for 8 courses. The high-dose Ara-c chemotherapy regimen (HAC) consisted of Ara-c (250 mg/m^2^ twice daily IV guttae, Day 1–5), VDS (3 mg/m^2^/day IV bolus, Day 1) and dexamethasone (6 mg/m^2^/day, IV or orally, Day 1–5), once every 4 weeks for 4 courses, and 4 courses of LAC regimen.

The maintenance therapy was performed for six months after the both regimens, which included VDS (3 mg/m^2^/day IV bolus, every 3 weeks), prednisone (40 mg/m^2^ /day orally, Day1–5, every 3 weeks), and 6-mercaptopurine (50 mg/m^2^ /day orally, daily). The disease activity score (DAS) was evaluated after four courses and eight courses of the induction treatment.

Treatment responses included nonactive disease (NAD), active disease-better (AD-B), active disease-stable (AD-S), active disease-worse (AD-W) according to the International LCH Study Group Criteria [9, 17]. DAS is evaluated according to Histiocyte Society criteria [18].

### Outcome

The primary outcome was response after four and eight courses of induction treatment. The secondary outcomes included the changes of DAS, event-free survival rate (EFS), OS and chemotherapy toxicity. Events were defined as disease progression, relapse, and death of activation of primary disease or toxicity, whichever came first. The patients without event were censored at the date of the last follow-up. Adverse events were evaluated according to Common Terminology Criteria for Adverse Events.

### Statistical analysis

Continuous variables were described as mean (standard deviation) or median (minimum and maximum) as appropriate, categorical variables as frequencies and percentage. Quantitative and qualitative variables were compared with the t test or the Wilcoxon rank test, and x-square test or Fisher exact test. Kaplan-Meier curves were used to estimate survival, and the differences in EFS and OS rates among different groups were compared by the log-rank test. All data were performed using IBM SPSS 25.0 software. A P-value < 0.05 was considered statistically significant.

## Results

### Patient characteristics

From January 2013 to December 2016, a total of 20 MS-RO + LCH patients treated with a combination of Ara-c, VDS and prednisone were included in the study, of whom 13 (65%) patients received the LAC regimen and 7 (35%) patients received the HAC regimen. The characteristics of the 20 patients were shown in Table 1. In the LAC and HAC groups, there were 5 (38.46%) and 6 (85.71%) males, and the median age was 1.67 (rage, 0.33–4.58) and 2.67 (rage, 1.42–9.83) years. They all received standard VDS-prednisone based first-line chemotherapy previously. In the LAC group, 11 (84.62%) patients were refractory to the first-line therapy and 2 (15.38%) had relapse. Among the 2 patients with relapse, 1 patient experienced reactivation of pituitary and bone after discontinuing the first-line therapy for 1 year and then received the LAC regimen. 1 patient experienced reactivation of bone after discontinuing the first-line therapy for 3 months. The patient restarted the VDS-prednisone based chemotherapy for 7 months and had progression of pulmonary and bone, and then was switched to LAC regimen. In the HAC group, 6 (85.71%) patients were refractory to the first-line therapy and 1 (14.29%) patient had relapse. The patient with relapse had reactivation of left cervical lymph nodes after discontinuing the first-line therapy for 6 months and received the HAC regimen.

**Table 1:** Characteristics of patients

Parameters	The LAC group n = 13		The HAC group n = 7
**General**			
Gender (male), n(%)	5 (38.46%)		6 (85.71%)
Median Age (year), (range)	1.67 (0.33–4.58)		2.67 (1.42–9.83)
DAS after initial therapy	2 (0–5)		2 (0–7)
Duration before diagnosis (W)	44.71 (6.14−120.43)		20.14 (8−156.43)
Follow-up time (years)	4.10 (0.92–6.67)		5.21 (2.68–6.23)
**Organ involvement and Laboratory tests**			
Liver involvement	11 (84.61%)		6 (85.761%)
Spleen involvement	7 (53.85%)		3 (42.86%)
Hematology dysfunction	3 (23.08%)		0 (0%)
AST (U/L)		34.3 (11.3-131.6)	53.8 (17.9–98.8)
ALT (U/L)		16.9 (9.2-226.3)	30.6 (10.1–82.5)
γ-GGT (U/L)		24 (12.2−182.1)	33.7 (9.1−479.7)

LAC, low-dose Ara-c chemotherapy; HAC, high-dose Ara-c chemotherapy; DAS, disease activity score; ALT, alanine aminotransferase; AST, aspartate aminotransferase; r-GGT, gamma glutamyltranspeptidase

### Response

**Fig. 1:**
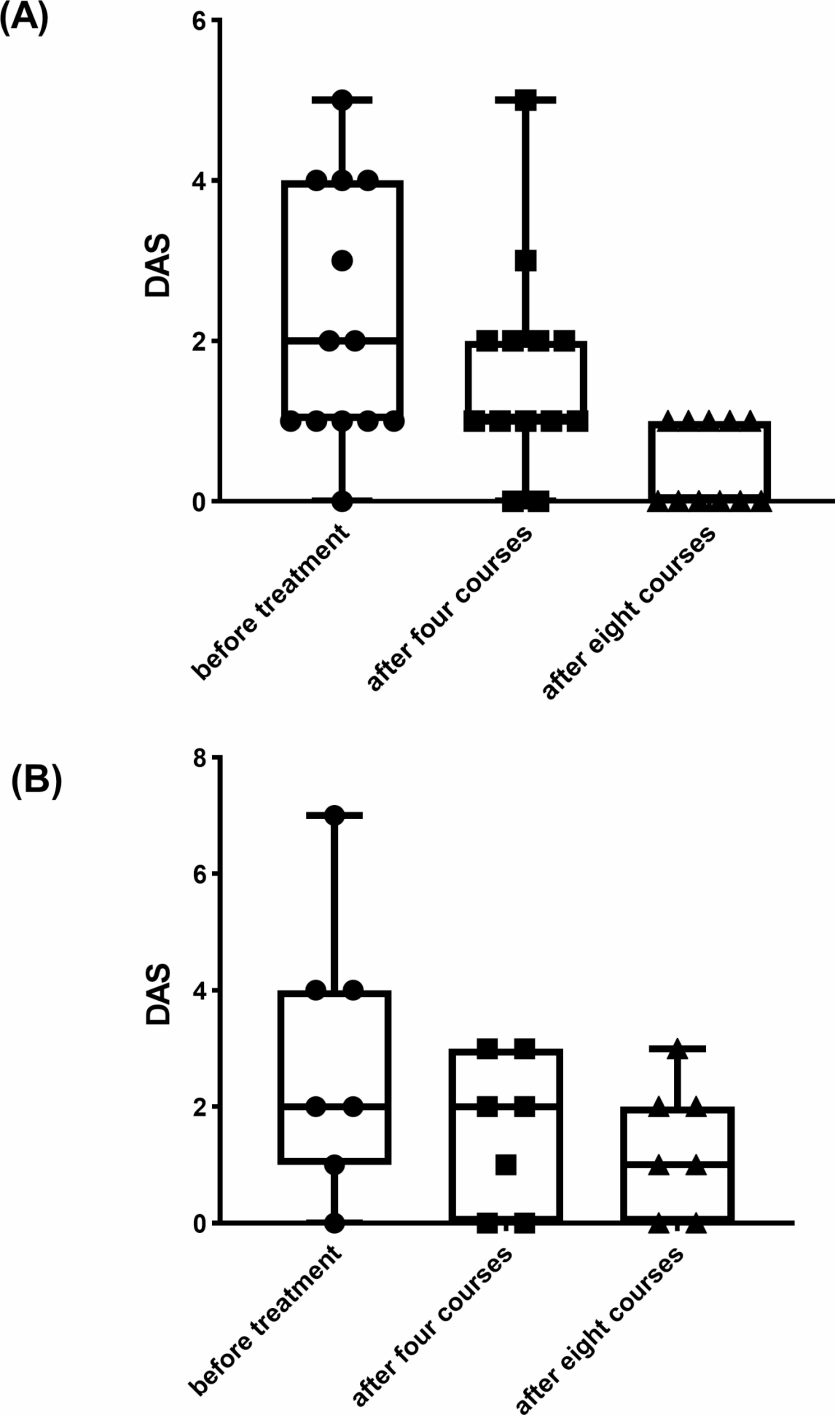
Changes of DAS before the treatment and after four and eight courses of the treatment in the LAC group **(A)** and HAC group **(B)**

The overall response rate (ORR) of the LAC group and the HAC group was 84.62% and 85.71% after four and eight courses of the treatment (Table 2). The numerical decrease of median DAS was observed both in LAC and HAC groups (Fig. 1).

**Table 2:** Response

	The LAC group n = 13	The HAC group n = 7	
**After four courses**			
NAD	0 (0%)	0 (0%)	
AD-B	11 (84.62%)	6 (85.71%)	
AD-S	1 (7.69%)	0 (0%)	
AD-W	1 (7.69%)	1 (14.29%)	
ORR	11 (84.62%)	6 (85.71%)	
**After eight courses**			
NAD	0 (0%)	0 (0%)	
AD-B	11 (84.62%)	6 (85.71%)	
AD-S	0 (0%)	0 (0%)	
AD-W	2 (15.38%)	1 (14.29%)	
ORR	11 (84.62%)	6 (85.71%)	

LAC, low-dose Ara-c chemotherapy; HAC, high-dose Ara-c chemotherapy; NAD,

nonactive active disease; AD-B, active disease-better; AD-S,active disease -stable;

AD-W, active disease-worse; ORR, overall response rate

### Outcome and survival

**Fig. 2:**
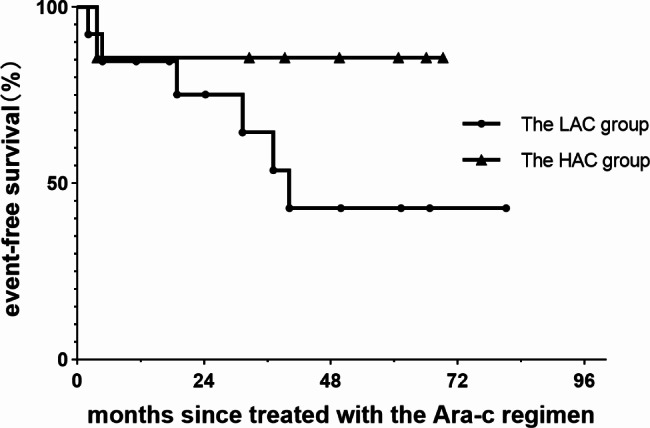
EFS of patients in the LAC and HAC groups

All patients were alive in the two groups during follow-up time. The median follow-up time in the LAC group and HAC group was 4.10 (rage, 0.92–6.67) and 5.21 (rage, 2.68–6.23) years. 3-year EFS of the patients treated with LAC and HAC regimen was 53.72% and 85.71% (Fig. 2). Among the 13 patients in the LAC group, 5 patients experienced reactivation: 3 during maintenance therapy and 2 after drug withdrawal, and 1 patient had disease progression after 4 courses of therapy. Among the 7 patients in the HAC group, 1 patient had disease progression after four courses of the therapy. He received targeted therapy but still had recurrent reactivation after the targeted drug reduced or withdrawal.

### Safety

The most frequent chemotherapy-related adverse event was myelosuppression in both the two groups. 53.85% (7/13) and 71.43% (5/7) of the patients experienced myelosuppression in the LAC and HAC group. Only 15.38% (2/13) and 28.57% (2/7) of the patients had Grade3/4 myelosuppression in the LAC and HAC groups. Most patients could recover from myelosuppression after blood component transfusion and recombinant human granulocyte colony stimulating factor. Pneumonia was also observed in 23.08% (3/13) and 28.57% (2/7) of the patients treated with the LAC and HAC regimen (Table 3).

**Table 3:** Adverse events

	The LAC group n = 13	The HAC group n = 7	
**Myelosuppression**			
< 3 Grade	5 (38.46%)	3 (42.86%)	
≥ 3 Grade	2 (15.38%)	2 (28.57%)	
total	7 (53.85%)	5 (71.43%)	
**Pneumonia**	3 (23.08%)	2 (28.57%)	

LAC, low-dose Ara-c chemotherapy; HAC, high-dose Ara-c chemotherapy

### Sequelae

Two patients presented with diabetes insipidus in the LAC group. In the HAC group, 2 patients presented with isolated hepatomegaly and hepatolithiasis, with normal liver enzymes.

## Discussion

LCH is a rare disease and the incidence ranges from 2.6 to 8.9 cases per.

million children, with the median age at diagnosis of 3 years [1]. The patients with MS-LCH who failed the front-line therapy had a high mortality, and who had recurrent reactivation had a higher rate of permanent sequelae [6, 19, 20]. The standard salvage therapy has not been established and the aim of our study was to analyze the efficacy and safety of a combination of Ara-c, VDS and prednisone in patients with refractory/relapse MS-RO + LCH.

In this study, we found Ara-c combined with VDS and prednisone might be an effective treatment for some refractory/relapse MS-RO + LCH patients, with low toxicity. Nucleoside analogs including Ara-c and 2-CDA have been demonstrated effective as a salvage therapy for refractory/relapse LCH patients, but there is no uniform treatment regimen for the dose and administration of the drugs. Varying doses of the combination of Ara-c and 2-CDA were used for patients with refractory/relapse MS-RO + LCH. In the phase II study of the Histiocyte Society (LCH-S-2005) for patients with refractory MS-RO + LCH, a salvage treatment based on a high-dose of the combination of Ara-c (1 g/m^2^/d×5 days) and 2-CDA (9mg/m^2^/d×5 days) resulted in an overall response rate of 92%, but had high toxicity, with the mortality rate of 15%, and all patients experienced Grade 4 hematological toxicity [9]. Rosso et al. designed a low-dose of the combination of Ara-c (100mg/m^2^/d×4 days) and 2-CDA (5mg/m^2^/d×5 days) in a series of nine patients with refractory MS-RO + LCH [10]. 78% (7/9) of the patients had disease improvement and had much less toxicity than the high-dose regimen, but most of the patients still had Grade3/4 anaemia and thrombocytopenia. Additionally, several studies have reported the administering of Ara-c or 2-CDA alone for patients with refractory/relapse LCH resulted in less toxicity. A low-dose 2-CDA (5mg/m^2^/d×5 days) is effective in the patients with refractory RO- LCH but the response rate did not exceed 22% in RO + LCH patients and all of them experienced reactivation, and the cumulative dose of 2-CDA should be limited to 200 mg/m^2^ because excessive doses were associated with potential development of myelodysplasia [21]. Therefore, the use of 2-CDA alone had limitations in the treatment of MS-RO + LCH. The regimen comprising Ara-c (100mg/m^2^/d×5 days), VCR and prednisone was used for the treatment of newly diagnosis LCH patients according to Japan LCH Study Group, with the response rate of 76.2% and EFS rate of 46.2% in MS-RO + LCH patients [11]. In an institutional series, the low-dose Ara-c (100 to 170mg/m^2^/d×3 to 5 days) with or without VCR and prednisone was used in recurrent LCH patients. 66.7% (4/6) of RO + LCH patients had disease improvement, with the 3-year OS rate of 100%, but the 3-year EFS rate was only 41% [12]. In our study, we also found Ara-c combined with VDS and prednisone might be effective for some refractory/ relapse MS-RO + patients. Most frequent adverse event was Grade 1/2 myelosuppression and none of the patients died of chemotherapy toxicity. But the low-dose Ara-c regimen had low EFS rate which was consistent with previous studies, and we found the patients treated with the high-dose Ara-c regimen had a numerically EFS rate than those treated with low-dose regimen.

A combination of low-dose Ara-c, VCR and prednisone has been used as a second-line treatment for patients with refractory/relapse MS-OR- LCH according to the LCH-IV protocol [1]. However, there is still a lack of data on the utility of the high-dose Ara-c for refractory/relapse MS-RO + LCH patients, and our study provided reference for clinical therapy. Due to the small sample size and the retrospective nature of our study, further studies are needed in the future.

BRAF inhibitors are an obvious therapeutic strategy for LCH, but chemotherapy still remains an important part of LCH therapy. BRAF V600E mutation was observed in 50–60% of patients and in up to 87% in the patients with MS-RO + LCH [22, 23]. Several studies have reported that the targeted therapy is effective and safe in children with refractory BRAF V600E positive RO + LCH [15, 24]. The patients with BRAF mutations, especially who complicate with HLH and are intolerant to chemotherapy, can benefit from targeted therapy [25, 26]. However, targeted therapy can not eradicate the neoplastic clone and most of the patients relapsed after drug withdrawal [15]. In a recent study, a patient treated with the combination of the BRAF inhibitor and chemotherapy based on Ara-c and 2-CDA achieved molecular remission and not relapsed after stopping the treatment [27]. Therefore, the combination of chemotherapy and targeted therapy may obtain a sustained remission and needs further studies.

The study has several limitations. First, it is a retrospective and nonrandomized study, and the number of cases is small. In addition, the median DAS of patients in our study was 2, which was lower than that in previous studies. So the regimen we reported might only be effective for some mild LCH patients. Therefore, further clinical trials are needed, and enroll more cases and more severe LCH patients.

## Conclusions

In conclusion, our study observed that a combination of Ara-c, VDS and prednisone was effective and safe for some patients with refractory/relapse MS-RO + LCH. The high-dose Ara-c regimen was associated with a numerically higher EFS rate. The high-dose Ara-c regimen might be a potentially superior regimen and needs further studies.

## Data Availability

The data that support the findings of this study are available on request from the corresponding author.

## Abbreviations

AD-B: Active disease-better
AD-S: Active disease-stable
AD-W: Active disease-worse
Ara-c: Cytarabine
DAS: Disease activity score
EFS: Event-free survival rate
HAC: High-dose Ara-c chemotherapy
HLH: Hemophagocytic lymphohistiocytosis
LAC: Low-dose Ara-c chemotherapy
LCH: Langerhans cell histiocytosis
MS: Multisystem
NAD: Nonactive disease
RO+: Risk organ involvement
ORR: Overall response rate
OS: Overall survival
VCR: Vincristine
VDS: Vindesine
2-CDA: Cladribine
